# Blue-Enriched White Light Improves Performance but Not Subjective Alertness and Circadian Adaptation During Three Consecutive Simulated Night Shifts

**DOI:** 10.3389/fpsyg.2020.02172

**Published:** 2020-08-18

**Authors:** Erlend Sunde, Torhild Pedersen, Jelena Mrdalj, Eirunn Thun, Janne Grønli, Anette Harris, Bjørn Bjorvatn, Siri Waage, Debra J. Skene, Ståle Pallesen

**Affiliations:** ^1^Department of Psychosocial Science, University of Bergen, Bergen, Norway; ^2^Department of Biological and Medical Psychology, University of Bergen, Bergen, Norway; ^3^Department of Clinical Psychology, University of Bergen, Bergen, Norway; ^4^Department of Global Public Health and Primary Care, University of Bergen, Bergen, Norway; ^5^Norwegian Competence Center for Sleep Disorders, Haukeland University Hospital, Bergen, Norway; ^6^Chronobiology, Faculty of Health and Medical Sciences, University of Surrey, Guildford, United Kingdom; ^7^Optentia, North-West University Vaal Triangle Campus, Vanderbijlpark, South Africa

**Keywords:** night work, alertness, performance, Fatigue, countermeasures, light, light emitting diode

## Abstract

Use of blue-enriched light has received increasing interest regarding its activating and performance sustaining effects. However, studies assessing effects of such light during night work are few, and novel strategies for lighting using light emitting diode (LED) technology need to be researched. In a counterbalanced crossover design, we investigated the effects of a standard polychromatic blue-enriched white light (7000 K; ∼200 lx) compared to a warm white light (2500 K), of similar photon density (∼1.6 × 10^14^ photons/cm^2^/s), during three consecutive simulated night shifts. A total of 30 healthy participants [10 males, mean age 23.3 (*SD* = 2.9) years] were included in the study. Dependent variables comprised subjective alertness using the Karolinska Sleepiness Scale, a psychomotor vigilance task (PVT) and a digit symbol substitution test (DSST), all administered at five time points throughout each night shift. We also assessed dim-light melatonin onset (DLMO) before and after the night shifts, as well as participants’ opinion of the light conditions. Subjective alertness and performance on the PVT and DSST deteriorated during the night shifts, but 7000 K light was more beneficial for performance, mainly in terms of fewer errors on the PVT, at the end of the first- and second- night shift, compared to 2500 K light. Blue-enriched light only had a minor impact on PVT response times (RTs), as only the fastest 10% of the RTs were significantly improved in 7000 K compared to 2500 K light. In both 7000 and 2500 K light, the DLMO was delayed in those participants with valid assessment of this parameter [*n* = 20 (69.0%) in 7000 K light, *n* = 22 (78.6%) in 2500 K light], with a mean of 2:34 (*SE* = 0:14) and 2:12 (*SE* = 0:14) hours, respectively, which was not significantly different between the light conditions. Both light conditions were positively rated, although participants found 7000 K to be more suitable for work yet evaluated 2500 K light as more pleasant. The data indicate minor, but beneficial, effects of 7000 K light compared to 2500 K light on performance during night work. Circadian adaptation did not differ significantly between light conditions, though caution should be taken when interpreting these findings due to missing data. Field studies are needed to investigate similar light interventions in real-life settings, to develop recommendations regarding illumination for night workers.

**Clinical Trial Registration:** www.ClinicalTrials.gov, identifier NCT03203538.

## Introduction

Night work is a common type of shift work (Eurofound, 2017), associated with a range of adverse health effects (Kecklund and Axelsson, 2016), as well as increased risk of occupational injury (Fischer et al., 2017). A major challenge with night work concerns increased sleepiness and deterioration of performance, especially vigilant attention, during the shifts (Lim and Dinges, 2008; Åkerstedt and Wright, 2009; Ganesan et al., 2019; Mulhall et al., 2019). The alertness and performance decrements reflect misalignment of the circadian timing system, as well as homeostatic build-up of sleep need due to extended time in wakefulness (Santhi et al., 2007; Borbely et al., 2016; Mulhall et al., 2019).

Circadian rhythms reflect processes displaying endogenous oscillations around 24 h. They play a key role in when we sleep and when we are awake, as well as in body temperature levels, secretion of several hormones (e.g., melatonin, cortisol) and in our cognitive performance throughout the day (Rajaratnam and Arendt, 2001). Circadian rhythms are controlled and coordinated by the pacemaker located in the suprachiasmatic nuclei (SCN), and the light-dark cycle provides the strongest cue for entraining the SCN to the external day and night (Roenneberg and Foster, 1997). Artificial light can mimic the effect of natural light and can consequently be used to entrain the circadian rhythm (Khalsa et al., 2003) and as such, if appropriately timed, can reduce circadian misalignment and provide better adaptation to a night work schedule (Smith et al., 2008).

In addition to circadian entrainment effects, light exposure can also elicit acute alerting responses, especially at night when alertness is normally low (Cajochen, 2007; Vandewalle et al., 2009; Souman et al., 2018). These nonvisual light responses have been shown to depend on several light characteristics (for a review see Prayag et al., 2019) including intensity (Cajochen et al., 2000; Zeitzer et al., 2000), exposure duration (Chang et al., 2012), and spectral distribution (Brainard et al., 2001; Thapan et al., 2001; Lockley et al., 2003; Cajochen et al., 2005; Lockley et al., 2006). In addition, there is individual variability in the responses to light exposure (Chellappa et al., 2017; Gabel et al., 2017; Phillips et al., 2019). Supported by classical rod and cone photoreceptors, the nonvisual light responses are mainly driven by intrinsically photosensitive retinal ganglion cells (ipRGCs), expressing the light-sensitive photopigment melanopsin, that project light information to the SCN and brain areas involved in sleep regulation, arousal, and attention (Perrin et al., 2004; Vandewalle et al., 2009; Warthen and Provencio, 2012). The power of light intensity in eliciting nonvisual responses such as alertness and circadian entrainment is well known (Cajochen et al., 2000; Zeitzer et al., 2000). However, as melanopsin is maximally sensitive to short-wavelength light around 460–490 nm (Bailes and Lucas, 2013), monochromatic blue and polychromatic blue-enriched light, especially at relatively low intensities, can also elicit larger nonvisual responses than light with longer wavelengths (Lockley et al., 2003; Cajochen et al., 2005; Lockley et al., 2006; Chellappa et al., 2011; Brainard et al., 2015).

Previous studies investigating nonvisual responses to nocturnal short-wavelength light have mainly been conducted in laboratory settings, applying monochromatic light, and carefully controlling the participants’ environment, posture, nutritional intake and previous light exposure (Brainard et al., 2001; Thapan et al., 2001; Lockley et al., 2003; Cajochen et al., 2005; Lockley et al., 2006). Similarly, a few recent laboratory studies have included nocturnal polychromatic blue-enriched light (Cajochen et al., 2019; Hanifin et al., 2019). Thus, more naturalistic studies are warranted, and only a few recent studies have investigated nonvisual responses of nocturnal blue-enriched light during night work (Motamedzadeh et al., 2017; Sletten et al., 2017; Kazemi et al., 2018). Light conditions used in previous studies vary, and different ways of administering light such as by goggles, spheres and/or light boxes may not be applicable in practical settings. The development of cost-effective light emitting diode (LED) technology, has provided new and flexible strategies for illumination of workplaces (Schubert and Kim, 2005). Standard ceiling mounted LED-luminaires can easily be installed and tuned to provide light of specific intensity (Sunde et al., 2020), and/or specific spectral distributions (Canazei et al., 2017). Thus, LED-based standard lighting set-ups need to be investigated in order to elucidate if these light sources can sustain performance during night work. To the authors’ knowledge, only two previous studies have investigated nonvisual responses to lighting administered by ceiling-mounted LEDs during simulated night work (Canazei et al., 2017; Sunde et al., 2020). In one previous study from our research group (Sunde et al., 2020), bright light (∼900 lx, 4000 K) improved alertness and performance compared to standard light (∼90 lx, 4000 K), while another study by Canazei et al. (2017) found that varied reduced portions of short-wavelength light (2166–4667 K, ∼150 lx) did not impact alertness and performance during night shifts.

In the present study (ClinicalTrials.gov: NCT03203538) we investigated how a standard LED-based polychromatic blue-enriched white light (7000 K; ∼200 lx), compared to warm white light (2500 K) of similar photon density (∼1.6 × 10^14^ photons/cm^2^/s), affected subjective alertness and performance on attention tests during three consecutive simulated night shifts, as well as circadian adaptation to the night work schedule. We also investigated participants’ opinion of the lighting conditions and its feasibility for work. To ensure transferability to real-life settings, we employed relatively high illuminance (∼200 lx, at eye level in the direction of gaze) compliant with European standards for offices (CEN, 2011), as well as putting minimal restraints on participants during their spare time away from the laboratory night shifts. We hypothesized that three consecutive night shifts with 7000 K light would increase alertness and performance during shifts, and lead to a greater phase delay of the circadian rhythm hastening adaptation, compared to 2500 K light.

## Materials and Methods

### Participants

All participants were between 19 and 30 years and reported good to excellent health; no current or recent history of psychiatric-, neurological-, cardiovascular-, lung-, and/or sleep diseases/disorders; no medication use (except contraceptives); no eye disease and no color deficiency according to the 17-plate Ishihara Test for Color Deficiency. Female participants were not pregnant or breastfeeding. Participants were not engaged in night work and had no transmeridian travel in the month prior to and/or during the study period and were not extreme chronotypes according to the short Morningness-Eveningness Questionnaire (Adan and Almirall, 1991). Participants reported habitual sleep duration of 6–10 h and habitual wake time between 06:00 and 10:00 h. Participants had to refrain from alcohol use for 3 days prior to and during the simulated night shifts; caffeine use 1 week prior to and during the night shifts; and tobacco use at least 2 h prior to and during the simulated night shifts.

Participants were mainly students invited via mass e-mail and flyers/information at the University of Bergen. Prior to enrolment participants were screened by an online survey to ensure that they were eligible. A total of 33 (10 males) pre-screened individuals attended an enrolment session at the laboratory 3 days prior to the first simulated night shift. Written informed consent was obtained before participants completed a set of questionnaires (demographics) and performed a practice sequence comprising a cognitive test battery (see section “Laboratory Procedure”). Participants were compensated for their participation. The study was conducted according to the Declaration of Helsinki.

Of the 33 enrolled participants, two withdrew before the first night shift and one participant was excluded from both study periods due to wake times after 10:00 h and/or sleep duration < 6 h during the three baseline sleep periods/nights at home (see section “Design and Procedure”). Three participants had their first study period excluded, one due to illness, and two due to wake times after 10:00 h during baseline sleep. The final data set comprised 30 (10 males) participants (Table 1) with 29 (9 males) completing the night shifts in 7000 K light, and 28 (8 males) completing the night shifts in 2500 K light. A total of 27 (7 males) participants had valid data included for all six night shifts.

**TABLE 1:** Descriptive characteristics of the participants, and baseline sleep measured with actigraphy.

N total (males)	30 (10)	
Age [Mean (*SD*)]	23.3 (2.9)	
Body mass index [Mean (*SD*)]	23.2 (3.0)	
**Self-reported health (%)**		
Excellent	30.0	
Very good	53.0	
Good	17.0	
**Short-MEQ (%)**		
Moderately morning type	10.0	
Neither type	60.0	
Moderately evening type	30.0	

	**7000 K light (*n* = 29) Mean (*SD*)**	**2500 K light (*n* = 28) Mean (*SD*)**

**Baseline sleep (hh:mm)**		
Lights off	23:56 (1:11)	23:54 (0:57)
Sleep onset latency	0:17 (0:16)	0:14 (0:12)
Wake time	08:11 (0:55)	08:28 (1:15)
Time in bed	8:21 (1:10)	8:35 (1:18)

*Short-MEQ, short Morningness-Eveningness Questionnaire. Baseline sleep: average for three nights prior to the first night shift (including one night with scheduled saliva sampling until 1 h after usual bedtime). There were no significant differences between the light conditions (p > 0.05).*

Female participants reported their last menses onset and their usual menstrual cycle length. Using similar procedures as Vidafar et al. (2018), the menstrual phase (follicular, luteal) during the study periods was estimated. Three participants were in different menstrual phases during the two study periods, hence the vast majority (*n* = 17) were in the same menstrual phase during both study periods.

### Design and Procedure

**FIGURE 1:**
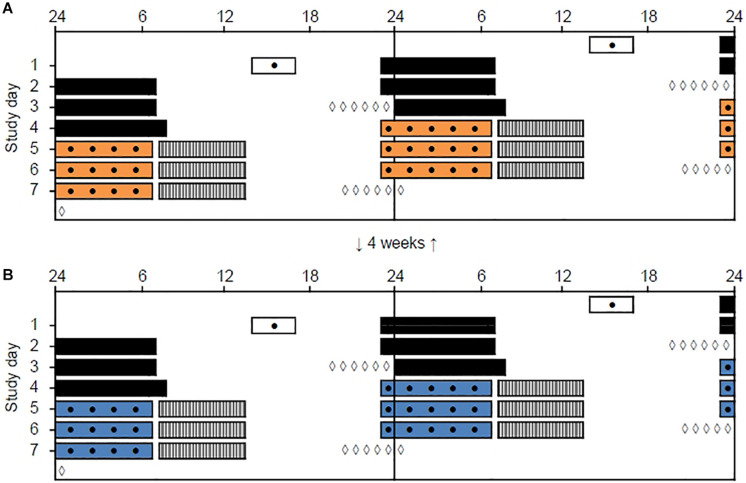
Double-raster plot of the simulated night work protocol. Clock hour is indicated on the *x*-axis and study day on the *y*-axis. The night work protocol included two study periods with three simulated night shifts (from 23:00 to 06:45 h) performed in a laboratory with different lighting conditions. **(A)** 2500 K light. **(B)** 7000 K light. The study periods were separated by 4 weeks and the order of conditions was counterbalanced. White bars indicate enrollment and practice session (before the first study period only) in the laboratory. Black bars indicate assumed baseline sleep at home. Colored bars indicate night shifts in the laboratory. Gray hatched bars indicate assumed daytime sleep at home. Black dots indicate primary test bouts including the Karolinska Sleepiness Scale (KSS), a Psychomotor Vigilance Task (PVT), and a Digit Symbol Substitution Test (DSST). White diamonds indicate salivary dim-light melatonin sampling at home.

The study was conducted from January to April 2018 and included two study periods, separated by 4 weeks, each containing three consecutive simulated night shifts (23:00–06:45 h) performed during a weekend (Friday evening to Monday morning) in a laboratory (see Figure 1). The study was conducted at a latitude (∼60°N) and at a time of year with relatively limited daylight exposure in the hours before and after the night shifts. Participants were allocated into four groups of 7–9 participants, and a counterbalanced crossover design with repeated measurements was employed. Thus, each participant performed night shifts under both light conditions, with about half of the participants starting in the 7000 K light condition and the other half starting in the 2500 K light condition. Participants slept at home and were instructed to keep a regular sleep schedule prior to the first night shift in accordance with their habitual sleep timing. The baseline sleep 3 days prior to the first night shift (Table 1) was monitored by sleep diaries and actigraphy to ensure that participants did not “turn night into day” before starting the simulated night work period. Bedtime on Thursday evening was not habitual due to hourly saliva sampling for estimation of dim-light melatonin onset (DLMO), which lasted until 1 h after usual bedtime. Napping was allowed before the night shifts, but not after 20:00 h and/or longer than 2 h. After completing the night shift in the laboratory participants went home to sleep *ad libitum* and with no restrictions concerning other activities, before meeting at the laboratory to complete the next night shift.

Sleep diaries and actigraphy indicated that napping was similar across study conditions. In 7000 K light, 18, 12, and 8 participants napped prior to the first, second, and third night shift, respectively. In 2500 K light, 14, 12, and 11 participants napped prior to the first, second, and third night shift, respectively. The duration of napping across conditions was also similar, with an overall mean napping duration of 1:14 (*SD* = 0:36) h and 1:21 (*SD* = 0:41) h in 7000 and 2500 K light, respectively. Most participants’ napping behavior was consistent for both study periods, and in terms of differences in napping between the light conditions, counterbalancing ensured that napping was very similar for both light conditions.

#### Laboratory and Light Exposure

**FIGURE 2:**
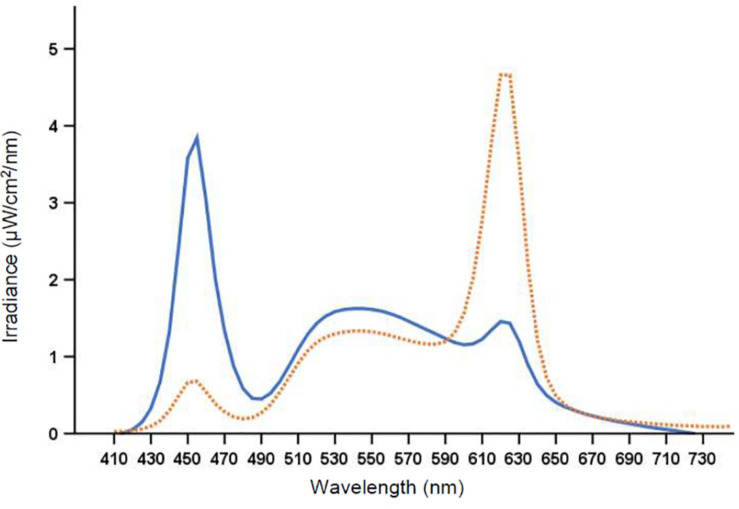
Spectral distribution of the 7000 K light (solid line) and the 2500 K light (dotted line).

The laboratory (30 m^2^) had no windows and the temperature was kept constant at ∼22°C. There were nine workplaces, each separated by partition walls, with identical desktop computers and screens fitted with a filter (Metolight SFG-10; Asmetec, Germany) blocking all light wavelengths < 520 nm. The laboratory was equipped with 20 ceiling mounted LED-luminaires (Modul R 600 LED CCT/RGB MP; Glamox Luxo Lighting AB, Sweden). Participants were exposed to polychromatic full-spectrum light with a color temperature of ∼7000 and ∼2500 K, respectively. The photopic illuminance was ∼200 lx in the vertical plane at eye level (∼600 lx in the horizontal plane), with similar photon density (∼1.6 × 10^14^ photons/cm^2^/s) for both light conditions. The color rendering index (*R*_a_) was > 80, and both light conditions were compliant with European standards for most interior areas, e.g., offices (CEN, 2011). Figure 2 shows the average spectral distribution of the light conditions measured at each workplace in the vertical plane, at eye level while seated (120 cm from the floor), using a spectroradiometer (GL Spectis 1.0 T Flicker; GL Optic, Poland). The photometric information of the light conditions is reported in Table 2, estimated using the Lucas et al. (2014) toolbox.

**TABLE 2:** Lighting parameters (380–780 nm inclusive) for nine workplaces.

	7000 K light Mean (*SD*)	2500 K light Mean (*SD*)
Correlated color temperature (K)	6953 (260)	2455 (43)***
Irradiance (μW/cm^2^)	61 (6)	55 (5)*
Photon flux (photons/cm^2^/s)	1.65 × 10^14^ (1.55 ×‘10^13^)	1.61 × 10^14^ (1.37 × 10^13^)
Photopic illuminance (lx)	197 (19)	206 (18)
**Human retinal photopigment weighted illuminance (α-opic lx)**		
Cyanopic	220 (23)	40 (4)***
Melanopic	192 (19)	86 (8)***
Rhodopic	195 (19)	113 (11)***
Chloropic	196 (19)	160 (54)***
Erythropic	190 (18)	206 (18)

Light measured 120 cm from the floor in the vertical plane. Values calculated according to the Lucas et al. (2014) toolbox. *p < 0.05; ***p < 0.001, compared to 7000 K light.

#### Laboratory Procedure

The simulated night shifts started at 23:00 h with a 30 min preparation and adaptation period in the laboratory. At 23:30 h the first of five repeated main test bouts (23:30, 01:00, 02:30, 04:00, and 05:30 h) commenced. Each test bout lasted ∼20 min and included the Karolinska Sleepiness Scale (KSS) (Åkerstedt and Gillberg, 1990), a computerized Psychomotor Vigilance Task (PVT) (Dinges and Powell, 1985), and a computerized Digit Symbol Substitution Test (DSST) (Jaeger, 2018). During testing participants were seated at their designated workplace and wore noise canceling headsets (BOSE QuietComfort 25, BOSE Corporation, United States) to ensure undisturbed performance. Between the main test bouts participants performed other tests and had breaks allowing quiet activities such as reading and conversation. A researcher was present throughout the night shifts to ensure adherence to the protocol. Water was available *ad libitum* during the night shift, and at about 02:00 h and 05:00 h a small standardized meal/snack (∼200 kcal) was provided.

#### Alertness and Performance Measures

The KSS assesses subjective alertness/sleepiness (Åkerstedt and Gillberg, 1990), and was completed at the beginning and end of each test bout with participants indicating their current level of sleepiness on a 9-point Likert scale ranging from 1, “very alert,” to 9, “very sleepy, fighting sleep, strenuous to keep awake.” We analyzed the average KSS rating for each test bout as a measure of the participants’ subjective alertness level.

The PVT assesses the ability to sustain attention and is a sensitive measure for detecting sleep loss and sleep deprivation effects (Lim and Dinges, 2008; Basner and Dinges, 2011). The PVT shows minor aptitude and learning effects and is thus suitable for repeated administration (Lim and Dinges, 2008). A 10 min version was used in the present study, and participants were instructed to respond with their dominant hand on the space bar when presented with a visual stimulus (a counting timer) on the screen. The interstimulus interval varied randomly from 2 to 10 s including 1 s feedback on response time (RT) after each trial. If no response was given after 30 s, a sound was played to alert the participant before a new trial began. RTs < 100 ms was considered false starts. The mean number of trials per PVT was 95 (*SD* = 6). The primary outcome metrics comprised the mean 1/RT (reciprocal RTs) and the number of lapses (RTs ≥ 500 ms) as suggested by Basner and Dinges (2011), but also the number of false starts (responses without stimulus), the fastest 10% RT (mean RT for the 10% fastest responses) and the slowest 10% 1/RT (mean 1/RT for the 10% slowest responses) were reported.

The DSST was administered directly following the PVT and provided a second performance measure sensitive to changes in cognitive function (Jaeger, 2018). Performance on the DSST improves with repeated administrations (Jaeger, 2018). To minimize these learning effects participants practiced the DSST once during the enrollment session 3 days prior to the first night shift, and the symbol-digit pairs were randomized for each administration. A 2 min version was used, and participants were instructed to pair nine randomly presented symbols with their corresponding digit as fast as possible without making errors. Target symbols were presented at the center of the screen and participants selected the corresponding digit from a symbol-digit array, displayed at the bottom of the screen continuously during the test. Participants used the mouse pointer to select the digits, and if no response was recorded after 5 sec, the next trial began. The mean number of trials per DSST was 81 (*SD* = 9), and the number of correct responses was used as the outcome metric.

#### Circadian Phase and Sleep

To provide a measure of circadian phase before and after each night work period, we assessed salivary DLMO on Thursday evening (“baseline DLMO”) and Monday evening (“final DLMO”). Hourly saliva sampling (six samples) was performed at home, using Salivette tubes (Sarstedt AG & CO, Germany), following a similar protocol previously described (Saxvig et al., 2013). Baseline DLMO sampling started 4 h before and lasted until 1 h after participants’ habitual bedtime, while final DLMO sampling was delayed by 1 h relative to the baseline DLMO sampling (Figure 1). To ensure dim light during sampling, participants were instructed to wear dark sunglasses (Uvex Athletic ISO 9001, Uvex Winter Holding GmbH & Co. KG, Germany) from 1 h prior to, and during, the whole sampling period. The lenses of these glasses reduce light intensity to <1.0% (Saxvig et al., 2013). Participants labeled the samples with clock time and stored them in their domestic refrigerator before delivery at the laboratory for storage at – 70°C.

Samples were assayed with enzyme-linked immunosorbent assay kit (EK-DSM, Bühlman Laboratories, Switzerland). The detection limit of the assay kit is 0.5 pg/mL and the functional sensitivity is 1.6–20.5 pg/mL. Samples were analyzed using a Wallac 1420 Multilabel counter (Perkin Elmer Inc., United States). The inter-assay variation was 13.4% for the low and 12.3% for the high control, with mean (*SD*) melatonin values of 5.3 (0.7) and 15.5 (1.9) pg/mL, respectively. The DLMO was defined as the time salivary melatonin concentration reached 4 pg/mL. Linear interpolation between adjacent samples was used to calculate DLMO, and if levels reached 3 pg/mL but not 4 pg/mL linear extrapolation was used (Keijzer et al., 2011). The difference between baseline DLMO and final DLMO was calculated to estimate the magnitude of the circadian phase shift after the three consecutive night shifts. In accordance with previously reported procedures (Smith et al., 2008), we also estimated the temperature minimum (Tmin) for each participant by adding 7 h to the DLMO. The phase angle after the night shifts was estimated based on the final DLMO and sleep onset and sleep offset of the daytime sleep after the third night shift. We excluded one participant’s phase angle for sleep onset, and one participant’s phase angle for sleep offset, due to social commitments interfering with the daytime sleep after the third night shift.

The circadian phase shift could only be calculated for a subset of the participants due to missing DLMO data. For 7000 K light, phase shifts were estimated for 20 (69.0%) of the 29 included participants, and for 2500 K light phase shifts were estimated for 22 (78.6%) of the 28 included participants. Five (16.7%) of the 30 participants had no valid phase shift estimates, while complete phase shift estimates (for both light conditions) were available for 17 (56.7%) participants. The main reason for missing DLMO data was that salivary melatonin concentration did not reach 3 pg/mL during DLMO sampling. For 7000 K light, two (6.9%) and six (20.7%) participants did not reach 3 pg/mL during baseline and final DLMO sampling, respectively. For 2500 K light, one (3.6%) and three (10.7%) participants did not reach 3 pg/mL during baseline and final DLMO sampling, respectively.

Sleep data were derived from wrist actigraphy (Actiwatch 2, Philips Respironics Inc., United States), worn on the non-dominant hand. Data were recorded in 30 s epochs with medium wake threshold sensitivity (40 counts/min), and time of inactivity for sleep onset and wake time set to 10 min (Actiware version 6.0, Phillips Respironics Inc., United States). As recommended (Smith et al., 2018), the start and end of rest intervals were manually scored based on a standardized inspection of the raw data and sleep diaries.

#### Evaluation of Lighting Conditions

To assess participants’ subjective evaluation of the lighting conditions, a questionnaire comprising a semantic differential scale adapted from Smolders and de Kort (2014, 2017) was used. The scale consists of nine adjective items on a 7-point scale. The first four items comprised the subscale “pleasantness” of the lighting (“unpleasant–pleasant,” “uncomfortable–comfortable,” “disturbing–not disturbing,” and “causing glare–not causing glare”) which was internally reliable with Cronbach’s α = 0.82, similar to that reported by Smolders and de Kort (2014, 2017). Four single items were used to assess the “clearness” (“unclear–clear”), “color” (“warm–cold”), “brightness” (“dim–bright”), and if the lighting was “activating” (“relaxing–stimulating”). One item was used to assess if the lighting was “suitable for work” (“unsuitable for work–suitable for work”). The evaluation of lighting conditions was completed at the beginning (∼23:15 h) of the first night shift and at the end (∼06:15 h) of the third night shift in both light conditions.

### Statistical Analysis

To analyze the KSS, PVT mean 1/RT, fastest 10% RT, slowest 10% 1/RT and DSST we used linear mixed models (LMM). Three LMMs for each of the dependent variables were modeled. In a random effect model *participant* was included as a random effect. In a main effects model, *light* (7000 K vs. 2500 K), *shift* (night 1, night 2, and night 3) and *time* (23:30, 01:00, 02:30, 04:00, and 05:30 h) were entered as fixed factors. In the interaction effects model *light* × *shift*, *light* × *time*, *shift* × *time*, and *light* × *shift* × *time* were entered. *Time* was treated as a fixed factor due to the fixed timing of the main test bouts, and that there were some protocol differences in tasks and occurrences between the test bouts (e.g., provision of a standardized snack). The LMMs were run with a maximum likelihood estimation, enabling comparison of the fit of successive models using -2 times the log of the likelihood (-2LL) to conduct a likelihood ratio test (*LRT*). The difference in -2LL between the random effect model and the main effects model, and between the main effects model and the interaction effects model was compared to the chi-square distribution. The degrees of freedom (*df*; with Satterthwaite approximation) used for comparison were equal to the difference in the number of parameters between the compared models. If there were significant interaction effects, but the *LRT* indicated poorer model fit, we trimmed the interaction effects model by removing non-significant interaction effects before conducting a second *LRT*, comparing the main effects model and the trimmed interaction effects model. The residuals from the final LMM were tested for normality with Shapiro-Wilk tests and by assessment of normality plots to ensure that assumptions were met. *F*-statistics are reported and pseudo *R*^2^ statistics (reduction in variance given as: % explained variance) were calculated for the models with the best fit. Multiple comparisons were performed using Bonferroni corrections to evaluate the difference between light conditions, shifts and time points. To visualize the findings for the KSS, PVT mean 1/RT and the DSST, we plotted the estimated marginal means (*EMM*) and the standard errors (*SE*) for the *light* × *shift* × *time* interaction, although the interaction effects model did not have the best fit. The PVT fastest 10% RT and slowest 10% 1/RT were plotted as a function of *light* and *time*, as the trimmed interaction effects model including the *light* × *time* interaction had the best model fit for the fastest 10% RT.

The number of PVT lapses and false starts were analyzed using generalized linear mixed models (GLMM) with a negative binominal distribution, as features of these count variables showed overdispersion and a distribution skewed toward zero. A corresponding modeling approach as described for the LMM (random effect model, main effects model and interaction effects model) was used for the GLMM. We employed Satterthwaite approximation for the *df* and robust estimation of standard errors (*SE*). The GLMM analyses use restricted maximum likelihood estimation, thus the *LRT* approach for testing model fit is not appropriate for comparing these models. The Akaike’s information criterion (*AIC*) and the Schwarz’s Bayesian criterion (*BIC*) were instead used for comparison of models (the model with the smallest *AIC*/*BIC* values was preferred).

To assess the effect of light condition on the magnitude of the circadian phase shift, we used an LMM with *participant* included as a random effect and *light* entered as a fixed factor. We used similar settings as described for the previous analyses and comparison with Bonferroni adjustments were made to evaluate the difference in effect between light conditions. Initial differences in baseline DLMO between the light conditions were investigated by paired samples *t*-tests. The difference in baseline DLMO was also investigated including only participants with complete DLMO estimates (*n* = 17) in both light conditions. To assess if baseline DLMO correlated with phase shift magnitude, we calculated Pearson’s product-moment correlation coefficients. We also assessed, using *t*-tests, the differences between light conditions for the baseline sleep and phase angle variables. Daytime sleep after the night shifts was analyzed with LMMs using similar procedures as described previously. *Participant* was included as a random effect and *light*, *shift*, and the *light* × *shift* interaction were entered as fixed factors.

To analyze the evaluation of light conditions, LMMs were used in a similar modeling approach (and settings) as described above. For each of the six dependent variables (pleasantness, clearness, color, brightness, activating, and work suitability), a random effect model with *participant* included as a random effect; a main effects model with *light* entered as a fixed factor; and a time-interaction effects model with *time* [time 1 (start of first shift) vs. time 2 (end of last shift)] and the *light* × *time* interaction entered as fixed factors were computed. *LRT*s were used to assess model fit (random to main effects model; *df* = 1, main to time-interaction effects model; *df* = 2), and multiple comparisons with Bonferroni corrections were conducted to investigate the difference between light conditions.

All statistical analysis was performed using IBM SPSS Statistics, version 25 (IBM Corp., United States).

## Results

### Karolinska Sleepiness Scale (KSS)

**FIGURE 3:**
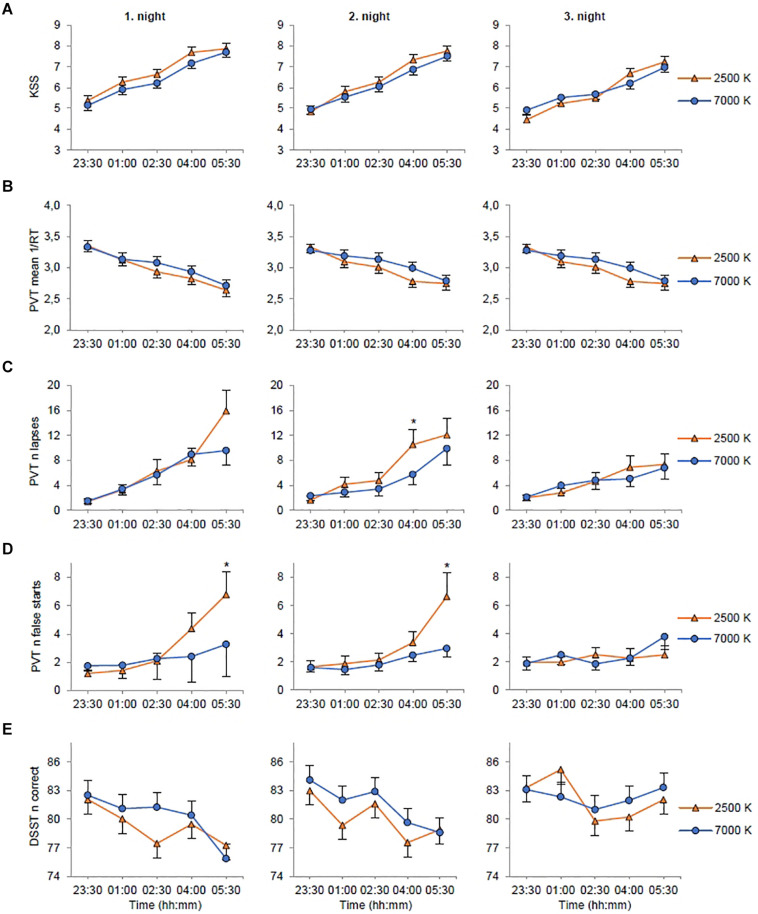
Estimated marginal means and standard error plotted as a function of light condition (2500 K vs. 7000 K light), night shift, and time of testing. **(A)** Rating on the Karolinska Sleepiness Scale (KSS). **(B)** Mean reciprocal response time (1/RT) on the Psychomotor Vigilance Task (PVT). **(C)** Number of lapses (RTs ≥ 500 ms) on the PVT. **(D)** Number of false starts (response without stimulus) on the PVT. **(E)** Number of correct responses on the Digit Symbol Substitution Test (DSST). *p < 0.05 between light conditions (only for variables with significant *light* × *night* × *time* interactions).

For KSS (Table 3) there were significant main effects of *light* with reduced sleepiness/increased alertness in 7000 K compared to 2500 K light; *shift* with increased alertness on night 2 (*EMM* = 6.29; *SE* = 0.16, *p* = 0.003) and night 3 (*EMM* = 5.84; *SE* = 0.16, *p* < 0.001) compared to night 1 (*EMM* = 6.59; *SE* = 0.16); and *time* with reduced alertness at 01:00 (*EMM* = 5.71; *SE* = 0.17, *p* < 0.001), 02:30 (*EMM* = 6.05; *SE* = 0.17, *p* < 0.001), 04:00 (*EMM* = 6.99; *SE* = 0.17, *p* < 0.001) and 05:30 h (*EMM* = 7.50; *SE* = 0.17, *p* < 0.001) compared to 23:30 h (*EMM* = 4.95; *SE* = 0.17). The main effects model had the best fit (*df* = 7, *LRT* = 462.53) and explained 32.3% of the variance in KSS scores. There were no significant interaction effects (Figure 3A).

**TABLE 3:** Alertness and performance estimates for the light conditions, and *F*-statistics for fixed factors.

	7000 K light	2500 K light	Light		Shift		Time		Light*Shift		Light*Time		Shift*Time		Light*Shift*Time	
	*EMM (SE)*	*EMM (SE)*	*F (df)*	*p*	*F (df)*	*p*	*F (df)*	*p*	*F (df)*	*p*	*F (df)*	*p*	*F (df)*	*p*	*F (df)*	*p*
KSS [1-9 (sleepy)]	6.15 (0.16)	6.32 (0.16)	**4.58 (1.834)**	**0.033**	**31.80 (2.825)**	**<0.001**	**138.05 (4.825)**	**<0.001**	2.10 (2.825)	0.123	1.63 (4.825)	0.165	0.54 (8.825)	0.824	0.47 (8.825)	0.892
PVT mean 1/RT	3.06 (0.08)	3.01 (0.08)	2.89 (1.812)	0.090	1.64 (2.807)	0.195	**61.25 (4.807)**	**<0.001**	2.15 (2.807)	0.117	1.71 (4.807)	0.146	1.57 (8.807)	0.129	0.38 (8.807)	0.931
PVT *n* lapses	4.39 (0.93)	4.86 (0.96)	0.42 (1.807)	0.519	1.54 (2.807)	0.214	**27.08 (4.807)**	**<0.001**	0.59 (2.807)	0.555	9.99 (4.807)	0.999	**3.48 (8.807)**	**0.001**	**2.95 (8.807)**	**0.003**
PVT *n* false starts	2.19 (0.34)	2.49 (0.41)	1.53 (1.807)	0.308	0.08 (2.807)	0.921	**10.48 (4.807)**	**<0.001**	2.40 (2.807)	0.092	**2.61 (4.807)**	**0.034**	1.73 (8.807)	0.089	**2.78 (8.807)**	**0.005**
PVT fastest 10% RT	257.99 (4.57)	259.24 (4.58)	1.52 (1.810)	0.283	**7.95 (2.807)**	**<0.001**	**40.80 (2.807)**	**<0.001**	0.21 (2.807)	0.812	**2.91 (4.807)**	**0.021**	1.02 (8.807)	0.421	0.55 (8.807)	0.821
PVT slowest 10% 1/RT	1.92 (0.09)	1.86 (0.09)	2.65 (1.815)	0.104	1.08 (2.807)	0.339	**61.29 (4.807)**	**<0.001**	2.64 (2.807)	0.072	0.79 (4.807)	0.534	1.64 (8.807)	0.110	0.62 (8.807)	0.760
DSST *n* correct	81.35 (1.14)	80.50 (1.14)	**4.82 (1.816)**	**0.028**	**14.83 (2.810)**	**<0.001**	**12.20 (4.810)**	**<0.001**	0.80 (2.810)	0.449	1.26 (4.810)	0.285	**4.37 (8.810)**	**<0.001**	1.30 (8.810)	0.239

**EMM, estimated marginal means. SE, standard error. KSS, Karolinska Sleepiness Scale. PVT, Psychomotor Vigilance Task. DSST, Digit Symbol Substitution Test. Estimates and statistics were calculated using linear- or generalized linear mixed models. Significant findings are indicated in bold.**

### Psychomotor Vigilance Task (PVT)

For mean 1/RT (Table 3) there were no significant main effects of *light* or *shift*, but there was a significant main effect of *time* with slower RTs at 01:00 (*EMM* = 3.14; *SE* = 0.08, *p* < 0.001), 02:30 (*EMM* = 3.04; *SE* = 0.08, *p* < 0.001), 04:00 (*EMM* = 2.91; *SE* = 0.08, *p* < 0.001) and 05:30 h (*EMM* = 2.78; *SE* = 0.08, *p* < 0.001) compared to 23:30 h (*EMM* = 3.31; *SE* = 0.08). The main effects model had the best model fit (*df* = 7, *LRT* = 218.68) and explained 9.9% of the variance in mean 1/RT. There were no significant interaction effects (Figure 3B).

For number of lapses (Table 3) there were no significant main effects of *light* or *shift*, but there was a significant main effect of *time* with more lapses at 01:00 (*EMM* = 3.37; *SE* = 0.73, *p* < 0.001), 02:30 (*EMM* = 4.84; *SE* = 1.07, *p* < 0.001), 04:00 (*EMM* = 7.32; *SE* = 1.31, *p* < 0.001) and 05:30 h (*EMM* = 9.84; *SE* = 1.79, *p* < 0.001) compared to 23:30 h (*EMM* = 1.79; *SE* = 0.40). There were also significant interaction effects of *shift* × *time* with fewer lapses at 04:00 (*EMM* = 5.92; *SE* = 1.31, *p* = 0.005) and 05:30 h (*EMM* = 7.05; *SE* = 1.53, *p* = 0.001) on night 3 compared to 04:00 h (*EMM* = 8.53; *SE* = 1.39) and 05:30 h (*EMM* = 12.35; *SE* = 2.15) on night 1; and *light* × *shift* × *time* (Figure 3C). The interaction effects model (*AIC, BIC* = 2717, 2722) had smaller *AIC/BIC* values than the main (*AIC, BIC* = 2741, 2746) and random (*AIC, BIC* = 2819, 2824) effects model.

For number of false starts (Table 3) there were no significant main effects of *light* or *shift*, but there was a significant main effect of *time* with more false starts at 05:30 h (*EMM* = 4.01; *SE* = 0.657, *p* = 0.001) compared to 23:30 h (*EMM* = 1.66; *SE* = 0.26). There were also significant interaction effects of *light* × *time* with fewer false starts at 05:30 h with 7000 K (*EMM* = 3.33; *SE* = 0.57, *p* = 0.040) compared to 2500 K (*EMM* = 4.83; *SE* = 0.93) light; and *light* × *shift* × *time* (Figure 3D). The interaction effects model (*AIC, BIC* = 2636, 2641) had smaller *AIC/BIC* values than the main (*AIC, BIC* = 2676, 2681) and random (*AIC, BIC* = 2880, 2884) effects model.

**FIGURE 4:**
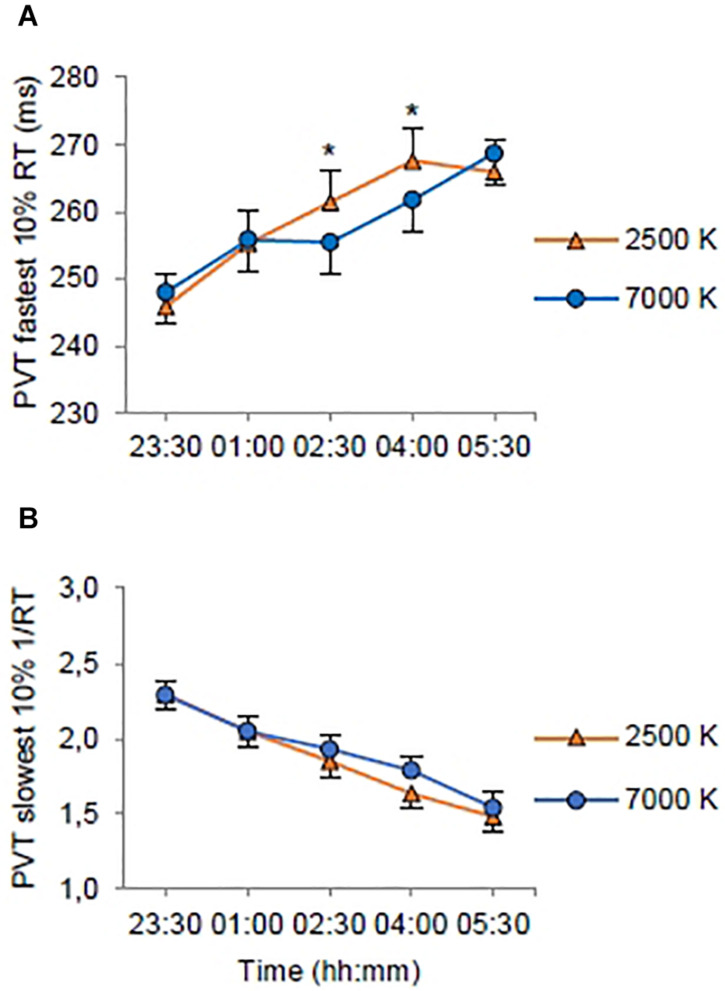
Estimated marginal means and standard error plotted as a function of light condition (2500 K vs. 7000 K light) and time of testing (all night shifts included). **(A)** Response times (RT) for the 10% fastest RTs on the Psychomotor Vigilance Task (PVT). **(B)** Mean resiprocal RTs (1/RT) for the 10% slowest RTs on the PVT. *p < 0.05 between light conditions.

On the fastest 10% RT (Table 3) there was no significant main effect of *light*, but there were significant main effects of *shift* with shorter RTs on night 2 (*EMM* = 258.08; *SE* = 4.61, *p* < 0.022) and night 3 (*EMM* = 256.18; *SE* = 4.61, *p* < 0.001) compared to night 1 (*EMM* = 261.58; *SE* = 4.61); and *time* with longer RTs at 01:00 (*EMM* = 255.60; *SE* = 4.67, *p* < 0.001), 02:30 (*EMM* = 258.45; *SE* = 4.67, *p* < 0.001), 04:00 (*EMM* = 264.69; *SE* = 4.67, *p* < 0.001) and 05:30 h (*EMM* = 267.33; *SE* = 4.67, *p* < 0.001) compared to 23:30 h (*EMM* = 247.00; *SE* = 4.67). There was a significant interaction effect of *light* × *time* (Figure 4A). The trimmed interaction effects model, including the *light* × *time* interaction, had a better model fit than the main effects model (*df* = 4, *LRT* = 11.56) and explained 6.0% of the variance in the fastest 10% RT.

On the slowest 10% 1/RT (Table 3) there were no significant main effects of *light* or *shift*, but there was a significant main effect of *time* with longer RTs at 01:00 (*EMM* = 2.05; *SE* = 0.09, *p* < 0.001), 02:30 (*EMM* = 1.89; *SE* = 0.09, *p* < 0.001), 04:00 (*EMM* = 1.71; *SE* = 0.09, *p* < 0.001) and 05:30 h (*EMM* = 1.51; *SE* = 0.09, *p* < 0.001) compared to 23:30 h (*EMM* = 2.29; *SE* = 0.09). The main effects model had the best model fit (*df* = 7, *LRT* = 217.73) and explained 13.2% of the variance in the slowest 10% 1/RT. There were no significant interaction effects (Figure 4B).

### Digit Symbol Substitution Test (DSST)

For the number of correct responses on the DSST (Table 3) there were significant main effects of *light* with more correct responses in 7000 K compared to 2500 K light; *shift* with more correct responses on night 3 (*EMM* = 82.24; *SE* = 1.15, *p* < 0.001) compared to night 1 (*EMM* = 79.74; *SE* = 1.15); and *time* with fewer correct responses at 02:30 (*EMM* = 80.67; *SE* = 1.18, *p* < 0.001), 04:00 (*EMM* = 79.89; *SE* = 1.18, *p* < 0.001) and 05:30 h (*EMM* = 79.33; *SE* = 1.18, *p* < 0.001) compared to 23:30 h (*EMM* = 83.02; *SE* = 1.18). There was also a significant interaction effect of *shift*× *time* with more correct responses at 02:30 h on night 2 (*EMM* = 82.26; *SE* = 1.32, *p* = 0.015) compared to night 1 (*EMM* = 79.36; *SE* = 1.32), at 01:00 h on night 3 (*EMM* = 83.78; *SE* = 1.32, *p* = 0.006) compared to night 1 (*EMM* = 80.57; *SE* = 1.32) and at 05:30 h on night 3 (*EMM* = 82.69; *SE* = 1.32, *p* < 0.001) compared to night 1 (*EMM* = 76.56; *SE* = 1.32). There were fewer correct responses at 02:30 (*EMM* = 79.36; *SE* = 1.32, *p* < 0.045) and 05:30 h (*EMM* = 76.56; *SE* = 1.32, *p* < 0.001) compared to 23:30 h (*EMM* = 82.29; *SE* = 1.32) on night 1, and at 04:00 (*EMM* = 78.60; *SE* = 1.32, *p* < 0.001) and 05:30 h (*EMM* = 78.75; *SE* = 1.32, *p* < 0.001) compared to 23:30 h (*EMM* = 83.55; *SE* = 1.32) on night 2. The interaction effects model had the best model fit (*df* = 22, *LRT* = 50.43) and explained 6.2% of the variance in the number of correct responses. However, there were no significant interaction effects of *light* × *time* or *light* × *shift* × *time* (Figure 3E).

### Circadian Phase and Sleep

**FIGURE 5:**
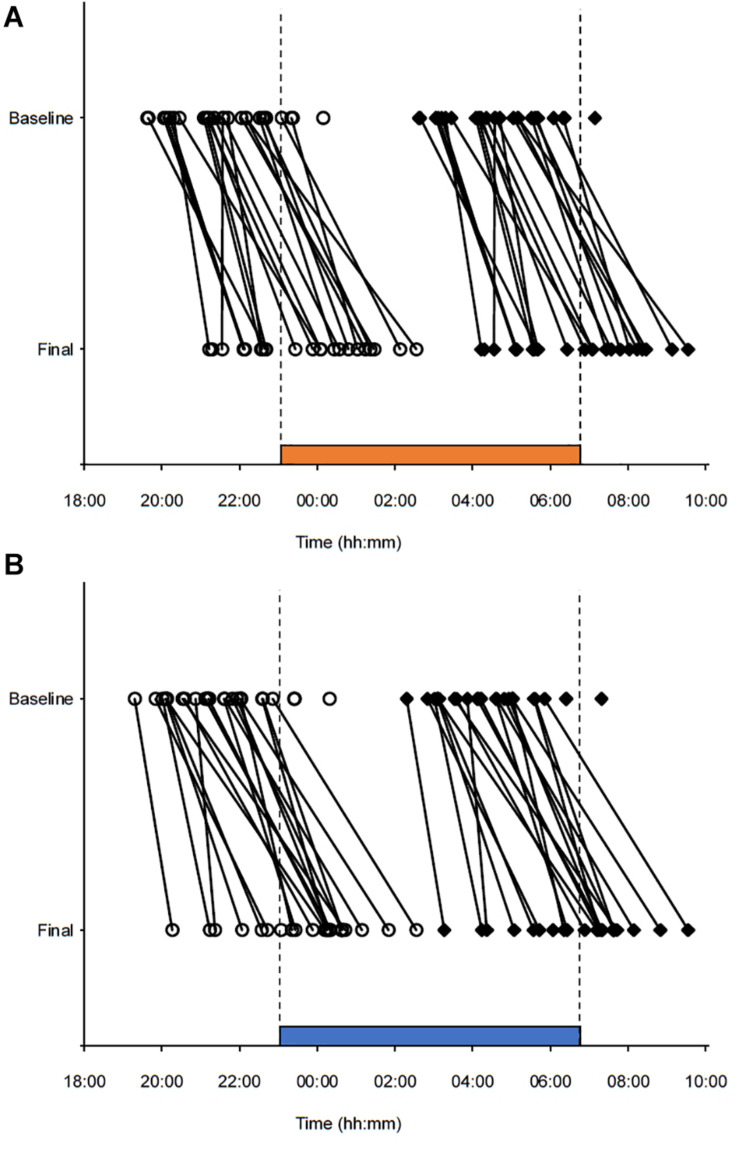
Phase markers for individual participants before (baseline) and after (final) three consecutive night shifts. **(A)** Night shifts in 2500 K light. **(B)** Night shifts in 7000 K light. Open circles indicate salivary dim-light melatonin onset (DLMO) for each participant. Filled diamond squares indicate estimated temperature minimum (DLMO + 7 h) for each participant. Lines are drawn between the baseline and final markers for each participant with complete baseline and final markers. The vertical dotted lines and the horizontal bars indicates the start and end times of the night shifts and light exposure.

All participants, except one in each light condition, showed a relatively robust circadian phase delay (≥30 min) after working three consecutive night shifts (Figure 5). For 7000 K light, the baseline DLMO (*n* = 26) ranged from 19:18 to 00:19 h, and for the final DLMO (*n* = 22) the range was 20:16–02:33 h. For 2500 K light, the baseline DLMO (*n* = 26) ranged from 19:37 to 00:09 h, and for the final DLMO (*n* = 23) the range was 21:13–02:33 h. In Table 4, DLMO and sleep statistics are provided for participants with complete data. Eleven participants had a larger phase delay after night shifts in 7000 K than in 2500 K light, while six participants showed an opposite effect. The LMM estimated mean phase delay of DLMO was 2:34 (*SE* = 0:14) h and 2:12 (*SE* = 0:14) h in the 7000 and 2500 K light conditions, respectively. However, there was no significant main effect of *light* (*F*_1,23_ = 1.58; *p* = 0.222). There was no significant difference in the initial timing of baseline DLMO before the night shifts across the two conditions (Table 4). Similar results were found when analyzing baseline DLMO for the 17 participants who had complete DLMO estimates in both light conditions (7000 K: *M* = 21:13; *SD* = 0:55, 2500 K: *M* = 21:27; *SD* = 1:03, *t*_16_ = 1.21; *p* = 0.243). The magnitude of phase shift and baseline DLMO did not correlate in either of the conditions (7000 K: *r* = 0.111; *p* = 0.640, 2500 K: *r* = 0.108; *p* = 0.632).

**TABLE 4:** Daytime sleep and circadian phase markers. Clock time (hh:mm).

	*n*	7000 K light Mean (*SD*)	2500 K light Mean (*SD*)
**Daytime sleep**			
Sleep onset	27	07:45 (0:28)	07:45 (0:32)
Sleep onset latency	27	0:06 (0:06)	0:05 (0:06)
Wake time	27	13:47 (0:57)	13:28 (1:00)
Sleep duration	27	6:01 (0:57)	5:43 (0:58)
**Circadian phase**			
Baseline DLMO	23	21:27 (1:10)	21:30 (1:06)
Final DLMO	18	23:54 (1:23)	23:36 (1:31)
Phase shift (delay)	17	2:43 (1:04)	2:12 (1:14)
**Phase angle**			
Phase angle sleep onset	16	7:36 (1:07)	7:47 (1:27)
Phase angle sleep offset	16	13:37 (2:35)	13:38 (1:55)

**Participants with complete data for each variable. Sleep variables derived from averaged actigraphy of the three daytime sleep periods after the night shifts. DLMO, dim-light melatonin onset. Baseline DLMO sampled in the evening one day prior to the first night shift; Final DLMO sampled in the evening on the day after the third night shift. Phase angle calculated as the time interval from final DLMO to sleep onset and offset, derived from actigraphy of the previous daytime sleep period (after the third night shift). There were no significant differences between the light conditions (p > 0.05).**

**FIGURE 6:**
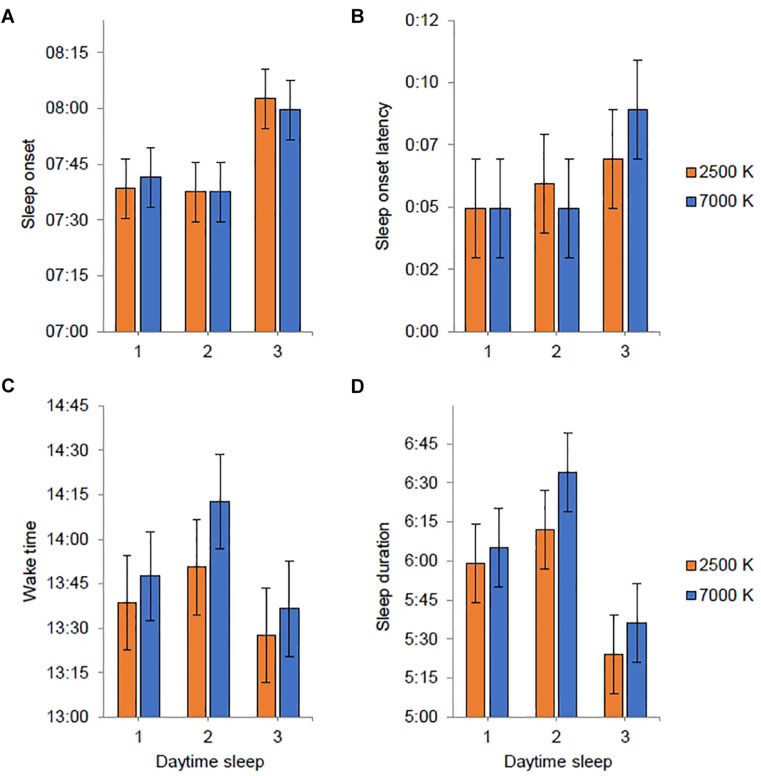
Estimated marginal means and standard error plotted as a function of light condition (2500 K vs. 7000 K light) and daytime sleep period (after night shift 1–3). Sleep variables were derived from actigraphy. Estimates are provided as clock time (hh:mm) for **(A,C)** and duration (h:mm) for **(B,D)**. No statistically significant differences between light conditions were found.

Participants had a mean daytime sleep duration after night shifts of 6:01 h in 7000 K light and 5:43 h in 2500 K light, which did not amount to a significant difference. Likewise, for the other daytime sleep variables, there were no significant differences between light conditions (Table 4). Also, the phase angle relationship for sleep onset and sleep offset did not differ significantly between the light conditions. In Figures 6A–D, estimates of daytime sleep after each night shift are provided. There was no significant main effect of *light* nor an interaction effect of *light* × *shift* for any of the sleep variables, and for sleep onset latency and wake time there were no significant effect of any of the fixed factors. For sleep onset there was a main effect of *shift* [*F*_(2, 139)_ = 6.14; *p* = 0.003] with later sleep onset for daytime sleep after night 3 (*EMM* = 08:01 h; *SE* = 0:06 h) compared to daytime sleep after night 1 (*EMM* = 07:40 h; *SE* = 0:06 h, *p* = 0.013) and night 2 (*EMM* = 07:38 h; *SE* = 0:06 h, *p* = 0.006). The main effects model explained 5.5% of the variance in sleep onset. For sleep duration there was a main effect of *shift* [*F*_(2, 139)_ = 8.23; *p* < 0.001] with shorter sleep duration for daytime sleep after night 3 (*EMM* = 5:31 h; *SE* = 0:12 h) compared to daytime sleep after night 1 (*EMM* = 6:03 h; *SE* = 0:12 h, *p* = 0.046) and night 2 (*EMM* = 6:24 h; *SE* = 0:12 h, *p* < 0.001). The main effects model explained 8.1% of the variance in sleep duration.

### Light Evaluation

For all the light evaluation items there was a significant main effect of *light* (Table 5). Participants evaluated 2500 K as more pleasant than 7000 K light, while 7000 K was evaluated as clearer, colder, brighter, more activating and more suitable for work than 2500 K light. Adding *light* as a factor significantly improved the model fit (*LRT*) compared with the random effects model for all measures (see explained variance in Table 5). For pleasantness, a significant interaction of *light* × *time* indicated that participants evaluated the 2500 K light as more pleasant than the 7000 K light only at time 2 (at the end of the third night shift). However, the *LRT* indicated that the time-interaction model did not significantly improve the model fit for any of the variables compared with the main effects model.

**TABLE 5:** Evaluation of light conditions estimates, and *F*-statistics for fixed factors.

	7000 K light	2500 K light	*Light*		*Time*		Light*Time		Variance (%) explained by *light*
	*EMM (SE)*	*EMM (SE)*	*F (df)*	*p*	*F (df)*	*p*	*F (df)*	*p*	
Pleasantness	4.64 (0.17)	5.13 (0.17)	**5.27 (1.88)**	**0.024**	0.13 (1.85)	0.725	**5.29 (1.85)**	**0.024**	**3.9**
Clearness	5.51 (0.23)	3.95 (0.23)	**41.27 (1.85)**	**<0.001**	0.21 (1.83)	0.651	1.08 (1.83)	0.301	**18.5**
Color	5.62 (0.22)	3.08 (0.22)	**75.19 (1.86)**	**<0.001**	1.58 (1.82)	0.213	0.41 (1.82)	0.525	**38.3**
Brightness	4.73 (0.19)	3.67 (0.19)	**29.10 (1.81)**	**<0.001**	1.82 (1.79)	0.181	0.49 (1.79)	0.484	**13.4**
Activating	5.11 (0.22)	3.24 (0.22)	**60.90 (1.84)**	**<0.001**	1.21 (1.82)	0.275	2.94 (1.82)	0.090	**26.8**
Suitable for work	5.72 (0.20)	4.21 (0.21)	**35.08 (1.86)**	**<0.001**	0.01 (1.3)	0.908	0.10 (1.83)	0.758	**20.4**

**EMM, estimated marginal means. SE, standard error. Pleasantness is a scale comprising four single items (pleasant, comfortable, disturbing and glary). All measures were derived from a 7-point semantic differential questionnaire completed at the beginning of the first night shift (time 1) and at the end of the last night shift (time 2). Estimates and statistics were calculated using linear mixed models. Adding the factors “time” and “light × time” interaction did not improve the model fit (Likelihood Ratio Test) for any of the variables. Significant findings are indicated in bold.**

## Discussion

In the current trial we applied novel strategies for administration of workplace lighting during three consecutive simulated night shifts, comparing blue-enriched (7000 K) and warm (2500 K) white light with similar photon density (∼1.6 × 10^14^ photons/cm^2^/s). As expected, subjective and behavioral alertness deteriorated throughout the night shifts. Blue-enriched light was more beneficial for alertness during night shifts compared to 2500 K light, but the differences were not clear-cut and mainly manifested as fewer PVT performance errors (lapses and false starts) at the end of the first and second night shift. Overall, subjective alertness was higher with 7000 K, compared to 2500 K light, but there were no significant interaction effects of light and time. Similarly, for the DSST there were more correct responses with 7000 K light, but no significant interaction of light and time. For the PVT mean 1/RT there were indications of shorter RTs with 7000 K light in the mid–late parts of the night shifts, albeit not statistically significant. However, for the fastest 10% RT, there were significantly shorter RTs with 7000 K light at 02:30 and 04:00 h, compared to 2500 K light. Altogether, our hypothesis that 7000 K compared to 2500 K light would increase alertness and performance during night shifts received partly support. For those with valid phase shift estimates (*n* = 20 (69.0%) and *n* = 22 (78.6%) in 7000 and 2500 K light, respectively], the melatonin rhythm was phase delayed after the night shifts. However, there was no significant difference in terms of circadian phase shifts between the two light conditions. Due to missing data the latter finding is inconclusive.

Monochromatic short-wavelength (i.e., blue) light has been shown to elicit alerting responses (Cajochen et al., 2005; Lockley et al., 2006). Although responses to polychromatic light may differ from responses to monochromatic light (Revell et al., 2010; Figueiro et al., 2018), polychromatic blue-enriched (6500 K; 40 lx) light has also been found to induce alertness compared to warm (2500 K; 40 lx) light in the evening (Chellappa et al., 2011). Likewise, we found evidence of alerting responses to blue-enriched light during simulated night work.

In a recent study, LED-based room lighting (∼150 lx) with high (4667 K), moderate (3366 K), and low (2166 K) color temperature during simulated night work, however, did not differentially impact perceived alertness and performance on a 25 min visual PVT (Canazei et al., 2017). The relatively lower color temperature employed in that study may explain the lack of differences between light conditions compared to the present study. Additionally, the PVT’s comparability with the version used in the present study is limited, as there were considerably fewer stimuli and substantially longer interstimulus intervals in the PVT applied by Canazei et al. (2017). Two other recent studies, using fluorescent light sources, assessed effects of nocturnal blue-enriched light on alertness and performance among real night workers (Motamedzadeh et al., 2017; Sletten et al., 2017). Similar to our findings concerning sleepiness, Sletten et al. (2017) found no differences between night workers exposed to blue-enriched light (17000 K; 89 lx) compared to standard light (4000 K; 84 lx). In addition, no differences regarding PVT performance was reported (Sletten et al., 2017). Sletten et al. (2017) did not use a crossover design, and between-subject differences may have confounded comparisons across conditions. The study by Sletten et al. (2017) also differed from the present study, as the light intervention commenced during one simulated night shift, following at least two consecutive night shifts at the participants’ usual occupation. Enhanced alertness with blue-enriched light was reported by Motamedzadeh et al. (2017), with lower subjective sleepiness among control room operators during 12 h night shifts with medium (6500 K) and high (17,000 K) blue-enriched light (∼350 lx), compared to standard light (4000 K; ∼350 lx). On a Continuous Performance Test, blue-enriched light did not affect errors of commission, but 6500 and 17,000 K light favored attention in terms of shorter RTs, and for 17,000 K light there were also fewer errors of omission (Motamedzadeh et al., 2017). Similarly, the present study found fewer PVT lapses (i.e., errors of omission) in the later parts of the first and second shift with 7000 K light, but also fewer PVT false starts (i.e., errors of commission). In addition, there were indications of shorter RTs with 7000 K light, as performance in the optimal (i.e., fastest 10% RT) domain of the PVT was improved with 7000 K light compared to 2500 K light. While slow PVT RTs (i.e., lapses) have been associated with activation of brain regions involved in the default mode network (i.e., resting state), performance in the optimal domain of the PVT has been associated with activation of regions involved in the fronto-parietal sustained attention network (Drummond et al., 2005), suggesting modulation by the nonvisual system via the blue light sensitive ipRGCs (Vandewalle et al., 2009; Chellappa et al., 2011; Warthen and Provencio, 2012). Contrary to the findings by Chellappa et al. (2011), we also found beneficial effects of blue-enriched light regarding PVT lapses, hence the nonvisual system may also modulate the default mode network related to slow RTs and lapses (Drummond et al., 2005).

None of the previous studies investigating blue-enriched light assessed performance using DSST. However, in a recent study we found that performance on the DSST during simulated night work may be improved by nocturnal bright (4000 K; ∼900 lx) compared to standard (4000 K; ∼90 lx) light (Sunde et al., 2020). The DSST is sensitive to change in cognitive function, and both attention and working memory are required for optimal performance (Jaeger, 2018). In the study by Motamedzadeh et al. (2017), a working memory test (n-back) revealed more correct responses with 17,000 K compared to 4000 K light, although Canazei et al. (2017) found no differences between light conditions on a working memory task.

Compared to the present study, the color temperature (17,000 K) was higher in the study by Sletten et al. (2017), yet the illuminance (89 lx) was substantially lower. Overall, the melanopic illuminance of the 7000 K light (192 lx) in the present study was higher than in the 17,000 K (129 lx) light in Sletten et al. (2017). However, the relative difference between the compared light conditions within the present study and the study by Sletten et al. (2017) was similar, with the blue-enriched light having about twice the melanopic illuminance as the control condition. Sletten et al. (2017) suggested that lack of differences between light conditions may reflect saturating light levels. Likewise, a highly controlled laboratory based study reported no differences in sleepiness between 9000 and 2800 K light (250 lx) and suggested that saturating light levels were used, and that spectral distribution is more important at lower light levels < 200 lx (Cajochen et al., 2019). Since in the present study higher illuminance was used than in Sletten et al. (2017), it cannot be ruled out that light saturation also influenced the current results. Still, in the study by Motamedzadeh et al. (2017), beneficial effects of blue-enriched light were found although the illuminance (∼350 lx) was even higher than in the present study.

It should be noted that previous studies concerning polychromatic light with different spectral distribution during night work (Canazei et al., 2017; Motamedzadeh et al., 2017; Sletten et al., 2017) have mainly used similar illuminance levels between the light conditions rather than being photon-matched. In the present study, however, the light conditions had similar photon density (∼1.6 × 10^14^), thus being the first blue-enriched light during night work study that has directly assessed the effectiveness of short-wavelength compared to long-wavelength light whilst correctly controlling for light intensity/photon density. Photon-matching was also used in a recent and highly controlled (e.g., participants were studied in a time-free environment for 7 days) laboratory trial (Hanifin et al., 2019), assessing alerting effects of nocturnal 6.5 h exposure to blue-enriched (17,000 K; 96 lx; 1.00 × 10^14^ photons/cm^2^/s) compared to standard (4000 K; 123 lx; 1.01 × 10^14^ photons/cm^2^/s) light. Subjective sleepiness was reduced with 17,000 K light compared to standard light, but 17,000 K light did not affect PVT measured RTs or lapses during light exposure (Hanifin et al., 2019). Hanifin et al. (2019) applied lower illuminance and a different spectral distribution compared to the present study. However, in the study by Hanifin et al. (2019) the melanopic illuminance in the standard (79 lx) light was only slightly lower than in the 2500 K (86 lx) light used in the present study, while the 17,000 K (133 lx) light in Hanifin et al. (2019) had lower melanopic illumination than the 7000 K (192 lx) light in the present study. Thus, it is a little surprising that we did not find stronger effects of 7000 K light on subjective alertness/sleepiness, as the mechanism is thought to be mediated by melanopsin expressing ipRGCs projecting to brain areas important for alertness and arousal regulation (Vandewalle et al., 2009; Warthen and Provencio, 2012). Still, compared to our study, much more rigorous control of participants’ exposure was taken, e.g., an ophthalmologic head holder was used to maintain a fixed head position and gaze, and light history was controlled with dim light and blindfolds prior to light exposure (Hanifin et al., 2019). As we found beneficial effects of 7000 K compared to 2500 K light on PVT measures, it is somewhat surprising that no effects were found during blue-enriched light exposure in the more controlled study by Hanifin et al. (2019).

In terms of polychromatic blue-enriched light, recent studies have not found greater phase delay with blue-enriched (17,000 K) compared to photon-matched standard (∼4000 K) light (Smith and Eastman, 2009; Hanifin et al., 2019), similar to the results in the present study. In the study by Smith and Eastman (2009), the blue-enriched light had a much higher illuminance (∼4000 lx) than in the current study and is thus not directly comparable. In the study by Hanifin et al. (2019), the light levels (1 × 10^14^ photons/cm^2^/s) were lower and more comparable to the current study. Although the current results are in line with the findings by Hanifin et al. (2019), the study protocols differ substantially. Importantly, in the current study the light exposure was kept constant throughout the night shifts, hence a portion of light exposure occurred after the estimated Tmin for most participants. In line with the phase response curve to light (Khalsa et al., 2003; Revell et al., 2012), and the fact that 7000 K light had about twice the melanopic illuminance than 2500 K light, it is likely that 7000 K light exposure after Tmin attenuated the phase delay to a larger degree than 2500 K light. Despite the fact that there were no significant differences in the phase delay magnitude between 7000 and 2500 K light, we observed beneficial effects of 7000 K light for PVT performance measures. Thus, blue-enriched light, as administered in the present study, may improve behavioral alertness without inducing larger phase delay than warmer light. This can be regarded as beneficial because circadian adaption to night work, implies that one later would also need to readapt to a day work schedule. Hence, for short-term night work (no more than 3 nights) it is not desirable to fully adapt during the night work period.

Participants evaluated 7000 K light as colder, brighter and more activating than 2500 K light, similar to a previous study of fluorescent light (6000 K vs. 2700 K light) during daytime office hours (Smolders and de Kort, 2017). Participants’ evaluation of 7000 K light as more activating than 2500 K light, contrasts the findings for the sleepiness and performance measures, where only minor advantages were found for 7000 K light. Thus, there may be some mismatch between subjective impressions of light effects and the actual test data on alertness and performance. In line with Smolders and de Kort (2017), participants evaluated 2500 K as more pleasant than 7000 K light. In contrast to lack of perceived differences during daytime office hours (Smolders and de Kort, 2017), 7000 K light was evaluated as clearer and more suitable for night work than 2500 K light. Thus, visual perception and appraisal of light conditions may differ during daytime and nighttime, possibly due to circadian and/or homeostatic processes also affecting subjective preferences for lighting. Noticeably, although there were differences in the evaluation of the pleasantness of the lights and their suitability for work, the participants evaluated both 7000 and 2500 K light as fairly pleasant and suitable for work.

Some limitations of the present study should be noted. Caution should be taken when interpreting the circadian phase shifting responses in the present study, as several participants (6 with 7000 K light and 3 with 2500 K light) did not reach the 3 pg/mL threshold during DLMO sampling after the night shifts, possibly because the light may have phase delayed DLMO beyond the fixed sampling time. Hence, it is possible that the 7000 K light caused a larger phase delay than could be measured, and the findings should be considered inconclusive. As for alertness and performance, saturating light levels may also explain lack of significant phase shift differences. In terms of external validity, several factors need to be considered when interpreting the present results. Most participants were females and given that male participants have shown greater responses to blue-enriched light (Chellappa et al., 2017), the results may not be generalizable to populations with a different sex distribution. Although menstrual phase is known to impact PVT performance (Vidafar et al., 2018), only three female participants were estimated to be in a different menstrual phase during the two study periods. However, as these were rough estimates based on self-report, we cannot completely rule out that menstrual phase may have affected the results. None of the participants had color vision deficiency according to the Ishihara test, but some females can be tetrachromatic, i.e., express a fourth cone pigment (Jacobs, 2018), and we do not know if such alterations occurred or may have affected the results. The crossover and counterbalanced design, however, reduced this impact. We only studied young healthy participants and, as age differences in the responses to blue-enriched polychromatic light have been reported (Gabel et al., 2017), the transferability to real-life settings including older workers is not clear. Another point is that the present study was conducted at a latitude and at a time of year where daylight exposure was limited in the hours before and after the night shifts. Thus, the generalizability to other latitudes and/or other seasons can be questioned, as prior light exposure may affect the alerting responses to light (Chang et al., 2013). In addition, reduced exposure to morning light after night shifts (e.g., during the commute home) can hasten circadian adaptation to night work (Smith et al., 2008). We did not tailor an individually adapted light intervention which could be beneficial considering the large variability in individuals’ circadian timing (Stone et al., 2018), and that individual differences in responses to light have been found (Phillips et al., 2019). However, in a real workplace, individually adapted light exposure using standard room lighting may be impractical, hence the current light intervention is generally more feasible and practical for workplaces. Still, it is now possible using modern LED technology to locally adjust the intensity and spectral distribution to facilitate desired nonvisual responses for individual workers. This should thus be explored in future studies. An issue regarding the use of LED-based blue-enriched light are potential hazards to the eyes, such as photochemical damage to the retina (Bullough et al., 2019), due to the blue-light exposure. However, reasonable foreseeable usage of LEDs is not expected to cause acute retinal damage, though possible long-term effects of exposure to new light sources need further research (International Commission on Non-Ionizing Radiation Protection, 2020).

In terms of study strengths we employed light conditions that are suitable for real-life application, and both conditions complied with European lighting standards for offices (CEN, 2011). Hence, compared to many previous studies of blue-enriched light (e.g., Chellappa et al., 2011; Hanifin et al., 2019), the current light conditions may be more suitable for a real-life workplace. We did not put requirements on participants’ behavior during spare time away from the laboratory, e.g., in terms of activities, sleep timing and light exposure, as we wanted to employ a protocol that was transferable to a real work schedule as much as possible which may be viewed as an asset in terms of generalizability. The light sources were photon-matched thus the effect of spectral composition was not confounded by differences in light intensity. Furthermore, light conditions were administered using standard ceiling mounted LED-luminaires that can easily be installed at a real workplace. In addition, the crossover design adjusted for individual differences that otherwise could have exerted a strong effect on the outcome variables.

## Conclusion

The present study indicated that standard LED-based polychromatic blue-enriched light (7000 K; ∼200 lx) compared to warm white light (2500 K) of similar photon density (∼1.6 × 10^14^ photons/cm^2^/s), had significant and beneficial, albeit minor impact on the alertness and performance decrements experienced during simulated night work. The circadian phase was delayed with both light conditions with no significant differences between conditions. However, the circadian phase shift findings were inconclusive due to missing data. Participants’ opinions of both light conditions were fairly positive, although 7000 K light was evaluated as more suitable for work, while 2500 K light was evaluated as more pleasant. In conclusion, LED-based blue-enriched light may facilitate alertness and performance during night work. More studies are needed to validate this conclusion, e.g., in different populations.

We encourage further research that makes full use of tunable LEDs, to elucidate lighting conditions favorable for night workers. Light interventions should be carefully planned to consider the various effects (e.g., subjective, cognitive and entrainment) of different light intensities and spectral distributions, and future studies in real workplaces are warranted to develop recommendations regarding illumination for night workers.

## Data Availability Statement

The raw data supporting the conclusions of this article will be made available by the authors, without undue reservation, to any qualified researcher.

## Ethics Statement

The study was reviewed and approved by the Regional Committee for Medical and Health Research Ethics, health region West, Norway (No. 2016/1903). The participants provided their written informed consent to participate in this study.

## Author Contributions

SP assisted by BB, JG, AH, SW, and ES: conceptualization. ES, JM, TP, ET, JG, BB, AH, SW, and SP: study design. ES, JM, TP, and DS: finalizing light conditions. ES, JM, TP, and ET: data collection. ES: data analysis. ES and SP: drafting the manuscript. ES, JM, TP, ET, JG, BB, AH, SW, DS, and SP: writing final draft. All authors contributed to the article and approved the submitted version.

## Conflict of Interest

The authors declare that the research was conducted in the absence of any commercial or financial relationships that could be construed as a potential conflict of interest.
